# First description of a multisystemic and lethal SARS-CoV-2 variant of concern P.1 (Gamma) infection in a FeLV-positive cat

**DOI:** 10.1186/s12985-022-01816-z

**Published:** 2022-05-26

**Authors:** Rodrigo Lima Carneiro, Jéssica Pires Farias, Josilene Ramos Pinheiro, Jackson Farias, André Carloto Vielmo, Alexander Birbrair, Aline Belmok, Fernando Lucas Melo, Bergmann Morais Ribeiro, Gepoliano Chaves, Paloma Oliveira Vidal, Wilson Barros Luiz, Jaime Henrique Amorim

**Affiliations:** 1 Department of Human Sciences, State University of Bahia, Salvador, BA Brazil; 2 Laboratory of Infectious Agents and Vectors (LAIVE), Center of Biological Sciences and Health, Federal University of Western Bahia, Rua da Prainha, 1326, Morada Nobre, Barreiras, Bahia CEP 47810-047 Brazil; 3 Multicentric Graduate Program in Biochemistry and Molecular Biology, Center of Biological Sciences and Health, Federal University of Western Bahia, Barreiras, BA Brazil; 4 Graduate Program in Biology and Biotechnology of Microorganisms, Department of Biological Sciences, State University of Santa Cruz, Ilhéus, BA Brazil; 5 Graduate Program in Investigative Pathology, Center of Biological Sciences and Health, Federal University of Western Bahia, Barreiras, BA Brazil; 6 Department of Dermatology, University of Wisconsin-Madison, Madison, WI USA; 7 Laboratory of Baculoviruses, University of Brasília, Brasília, DF Brazil; 8 Department of Pediatrics, University of Chicago, Chicago, IL USA; 9 Department of Pathology, Federal University of Minas Gerais, Belo Horizonte, Brazil; 10 Department of Radiology, Columbia University Medical Center, New York, USA

**Keywords:** COVID-19, Cats, SARS-CoV-2, Transmission, Multi-systemic viral infection

## Abstract

**Background:**

Phylogenetic studies indicate bats as original hosts of SARS-CoV-2. However, it remains unclear whether other animals, including pets, are crucial in the spread and maintenance of COVID-19 worldwide.

**Methods:**

In this study, we analyzed the first fatal case of a SARS-CoV-2 and FeLV co-infection in an eight-year-old male cat. We carried out a clinical evaluation and several laboratory analyses.

**Results:**

As main results, we observed an animal presenting severe acute respiratory syndrome and lesions in several organs, which led to the animal’s death. RT-qPCR analysis showed a SARS-CoV-2 as the causative agent. The virus was detected in several organs, indicating a multisystemic infection. The virus was found in a high load in the trachea, suggesting that the animal may have contribute to the transmission of the virus. The whole-genome sequencing revealed an infection by SARS-CoV-2 Gamma VOC (P.1), and any mutations indicating host adaptation were observed.

**Conclusion:**

Our data show that FeLV-positive cats are susceptible to SARS-CoV-2 infection and raise questions about the potential of immunocompromised FeLV-positive cats to act as a reservoir for SARS-CoV-2 new variants.

**Supplementary Information:**

The online version contains supplementary material available at 10.1186/s12985-022-01816-z.

## Background

Viruses from the *Coronaviridae* family are recognized for their ability to cross the species barrier and establish new host reservoirs of infection [1]. A disease (COVID-19, coronavirus disease 2019) caused by a new coronavirus (SARS-CoV-2, *Severe acute respiratory syndrome coronavirus 2*) emerged in 2019 and was declared as a pandemic on March 11, 2020. According to the World Health Organization, more than 460 million cases and more than 6.1 million deaths were attributed to COVID-19 since its initial report in late 2019 [2].

Phylogenetic studies indicate bats as original hosts of SARS-CoV-2. However, the species barrier jump from bats to humans is considered unlikely. The most probable hypothesis includes the existence of an intermediate host [3]. Such a scenario points out the importance of animals in the spread and even maintenance of COVID-19 worldwide. Such maintenance could be supported by events of reverse zoonosis, which was previously reported for dogs, cats, farmed minks, and zoo big felines [4, 5]. This may represent a threat to the health of pets, zoo, and wild animals. In addition, the replication of SARS-CoV-2 in animals represents a constant emergence risk of new variants with the potential to threaten human health and challenge the protective efficacy of vaccines currently available. This further emphasizes the need for a One Health approach to tackle SARS-CoV-2 re-emergence.

This study shows clinical and laboratory aspects of a fatal case of SARS-CoV-2 and FeLV (*Feline leukemia virus*) co-infection of a male cat. Detailed data presented here will be helpful to recognize and manage more susceptible animals to SARS-CoV-2 infection.

## Methods

### Ethics and animal experimentation

All handling procedures and experiments involving the animal were approved by the Committee for the Ethical Use of Animals in Research of the State Bahia University (no. 2021.005.0018150-89). The procedures involving the animal were carried out in accordance with the ethical and biosafety guidelines.

### Clinical evaluation

An eight-year-old male domestic cat of an undefined breed was admitted on April 10, 2021, with respiratory syndrome at a veterinary clinic in Barreiras, Bahia, Brazil. Data regarding respiratory aspects, temperature, heartbeat, weight, and other clinical aspects were kept during anamnesis. In addition, laboratory analyses were required due to the clinical condition of the animal.

### Blood cell count, biochemical and serological analysis

The blood sample was obtained by the jugular venous puncture and collected in test tubes with or without ethylenediaminetetraacetic acid (EDTA) at 2% (w/v), for hematological and biochemical/serological analyses, respectively. Complete blood cell counts (CBC) were performed using fresh blood samples with EDTA at 2% (w/v). Analyses were carried out using an automated Hematoclin 2.8 VET instrument (Bioclin, Brazil), according to the manufacturer`s instructions. In addition, serum levels of glucose, urea, creatinine, alanine and aminotransferase, alkaline phosphatase, and gamma-glutamyl transferase were determined using a Bio-100 semi-automated analyzer (Bioclin, Brazil), according to manufacturer`s instructions. Serological immunochromatographic tests for *Feline immunodeficiency virus* (FIV) and *Feline leukemia virus* (FeLV) were carried out using the FIV AC / FeLV AG COMBO VET FAST VET 013-1 (BIOCLIN, Brazil), according to the manufacturer’s recommended protocols. Moreover, a serological analysis based on Dot ELISA for the Feline infectious peritonitis virus (FIPV) was carried out using an ImmunoComb antibody test kit (VP DIAGNOSTICO, Brazil). The Dot ELISA analysis was carried out according to the manufacturer’s recommended protocols.

### Imaging diagnostics

Thoracic radiography procedure was performed using a digital imaging equipment (ECORAY, Korea) with 70 kV of potency and 1.2 milliampere seconds (mAs) as the radiographic technique. To evaluate respiratory conditions, right and left lateral and ventrodorsal projections were chosen. The entire procedure lasted 2 min.

### Necropsy and samples collection

The *postmortem* examination was performed immediately after the death, and the macroscopic changes were recorded using a digital camera. The body was placed in dorsal decubitus and the abdominal cavity was opened by medial incision, using the *linea alba* as a reference. To improve the exposure of pelvis and thorax, the hind limbs were disarticulated at the hip joint level and the forelegs folded down laterally, dissecting the skin and subcutaneous tissue of the submandibular and cervical regions. Afterward, costochondral disarticulation was performed in all fixation points of the ribs, and the cranial and caudal pubic branches were incised. After hyoid disarticulation, the trachea and esophagus were released between the cervical muscle fascia and the entrance to the thoracic cavity, and the monobloc was pulled so that it could be detached along with the entire thoracic extension to the diaphragm. Then, the diaphragm was sectioned in the dorsal semicircular portion, making a small incision in the right kidney and continuously sectioning the abdominal set parallel to the vertebral column up to the pelvic cavity. Finally, the pelvic cavity was contoured along with the external genitalia and anus so that the monobloc was released entirely from the cadaver.

Tissue samples of kidneys, lungs, heart, trachea, liver, intestines, and spleen were stored in 10% formaldehyde at room temperature or as fresh tissues at − 80 °C until analysis.

### Gross pathology and histopathology

Macroscopic evaluation of organs considered characteristics such as edema, congestion, discoloration, atelectasis, and consolidation. Tissues samples from kidneys, lungs, heart, trachea, liver, intestines, and spleen with a standardized size of 2.0 × 1.6 × 1.2 cm were fixed overnight in a 4% formaldehyde solution and buffered with sodium phosphate 0.1 M at pH 7.2. After dehydration with ethanol, the fragments were placed in xylol and then paraffined. Next, the samples were blocked using a TP 1020® sample blocker (LEICA, Germany) and microtomized using a RM 2255 rotary microtome (LEICA, Germany), according to the manufacturer`s instructions. After that, the samples were stained with hematoxylin and eosin.

### RNA extraction and reverse transcription quantitative PCR (RT-qPCR)

Nucleic acid extraction was carried out from tissue samples of the seven organs of the feline: lungs, trachea, spleen, liver, intestines, heart, and kidneys. Samples were prepared by adding 1 mL of Quik-Zol Trizol reagent (LUDWIG BIOTECNOLOGIA, Brazil) for each 100 mg of tissue and homogenized by vortexing. After this process, 250 µL of each sample was loaded onto columns of Cellco-Virus RNA + DNA Preparation Kit Spin (CELLCO BIOTEC, Brazil), and RNA was purified according to the manufacturer`s instructions.

The detection of SARS-CoV-2 was performed using the Allplex™ 2019-nCov Assay (SEEGENE, South Korea), according to the manufacturer`s instructions. Thermocycling was carried out in a QuantStudio 5 instrument (Applied Biosystems, USA) with a hold stage composed of a first step of 20 min at 50 °C, followed by a second step of 15 s at 95 °C. The PCR stage was composed of a first step of 15 s at 94 °C followed by a second step of 30 s at 58 °C, repeated 45 times.

### SARS-CoV-2 genome sequencing

SARS-CoV-2 genome from feline samples was recovered by amplicon tiling multiplex approach using nanopore sequencing. Briefly, RNA extractions (8 µL) from tissue samples were submitted to reverse transcription with LunaScript® (NEB, USA), following the manufacturer`s instructions. The obtained cDNA was used as template for SARS-CoV-2 genome amplification using Q5 Hot Start High-Fidelity DNA Polymerase (NEB, USA) 1200 bp amplicon "midnight" primer set [6] Thermocycling was composed of incubation for 30 s at 98 °C for denaturation, followed by 35 cycles of 98 °C for 15 s and 65 °C for 5 min for annealing and extension. PCR amplicons for pool 1 and pool 2 were combined for each sample and adjusted to a concentration of 5 -10 ng/µL. End-Prep reactions were performed with NEBNext® Ultra™ II End Repair/dA-Tailing Module (NEB, USA) and barcoded using ONT Native Barcoding Expansion kit (EXP-NBD104) (Oxford Nanopore Technologies, UK), according to manufacturers' protocols. The barcoded samples were then combined and purified using AMPure XP Beads (Beckman Coulter, USA) and loaded onto Oxford Nanopore MinION SpotON Flow Cells R9.4.1 (Oxford Nanopore Technologies, UK). High-accuracy base calling was performed using the Oxford Nanopore Guppy tool (Oxford Nanopore Technologies, UK).

Mapping, primer trimming, variant calling, and consensus assembly were performed with the artic-ncov2019 pipeline, using the Medaka protocol (https://artic.network/ncov-2019). The genome was assembled with at least 20 × coverage. Pango lineage was attributed to the newly assembled genome using the Pangolin v3.1.11 software tool (https://pangolin.cog-uk.io/).

### Phylogenetic analysis

Phylogenetic inferences were carried out comparing the SARS-CoV-2 genome obtained from the feline sample with a dataset of high-quality SARS-CoV-2 genomes available through GISAID sampled from December 2019 to Feb 2022 (Additional file 1: Table S1). Closely related sequences (no more than five mutations) were selected using AudacityInstant (GISAID) searches against the entire EpiCoV database. The final dataset consisted of 2334 human and cat samples from all the major WHO SARS-CoV-2 clades sampled mainly from Brazil and South America. The genomes were analysed using the NextStrain pipeline [7]. Briefly, the genomes sequences were aligned using MAFFT [8] and a maximum likelihood tree was inferred using IQ-Tree [9]. The ancestral reconstruction and timescale were estimated using augur and treetime [10]. The tree was rooted at Wuhan/WH01/2019 and Wuhan/Hu-1/2019 ancestor.

## Results

### Clinical evaluation and diagnosis of severe acute respiratory syndrome

**Fig. 1:**
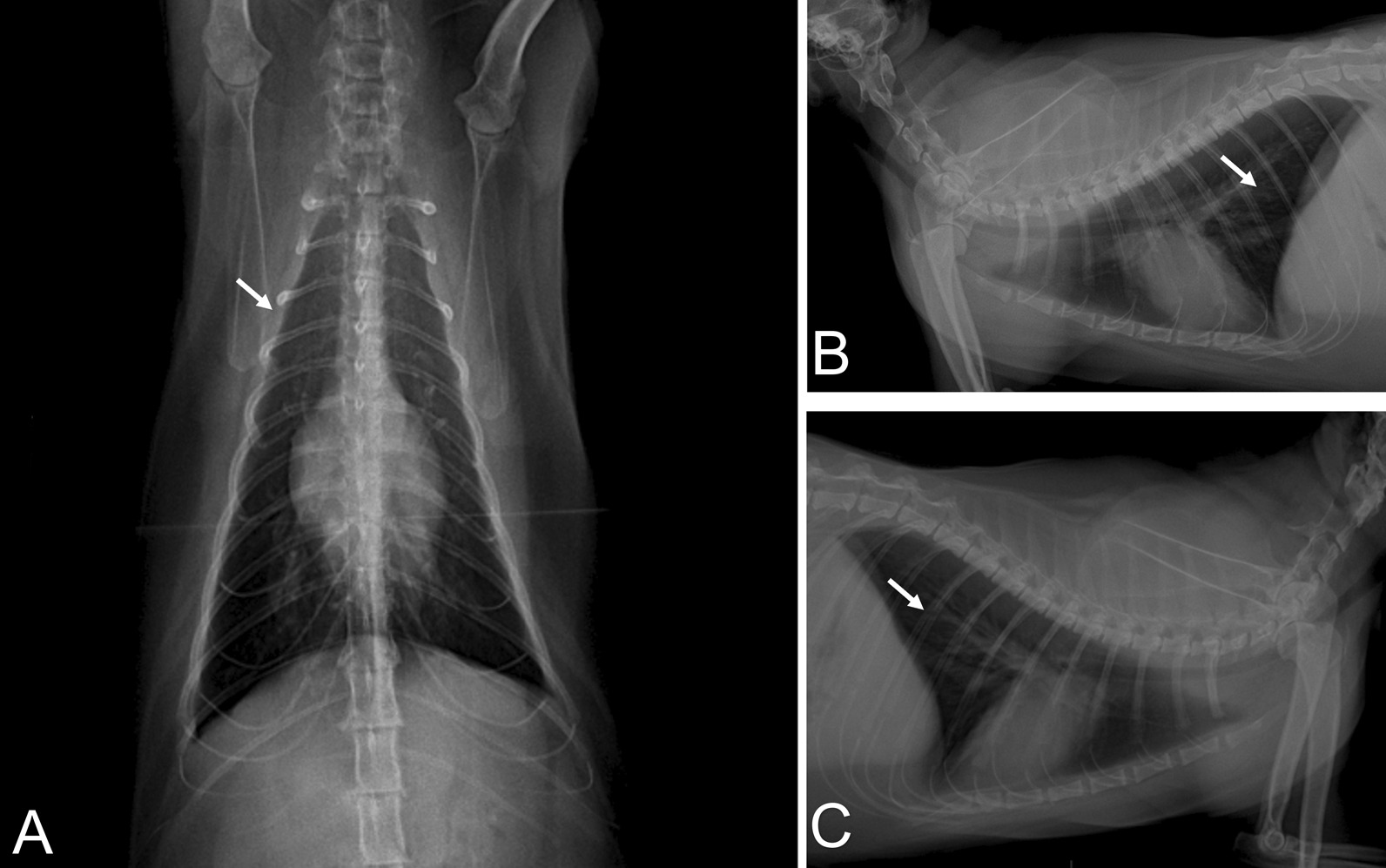
Radiographic views of ventrodorsal **a** lateral side (right and left, respectively), **b, c** increased radiopacity in the cranial and caudal lobes of both lungs accompanied by peribronchial infiltrate (white arrows) demonstrating incipient bronchitis

An eight-year-old male domestic cat of undefined breed was admitted at a veterinary clinic in Barreiras, Bahia, Brazil, presenting intense dyspnea, cyanotic mucous, and wheezing in the right hemithorax auscultation. The animal was previously exposed to its owner and more two persons, who were confirmed to be infected by SARS-CoV-2 (data not shown). Pet`s body temperature was considered normal for cats (38.6 °C). The oxygen saturation was shown to be 87%. In addition, thoracic radiography showed increased radiopacity in the cranial and caudal lobes of lungs, as well as peribronchial infiltrates compatible with incipient bronchitis, as shown by radiographic views of ventrodorsal (Fig. 1a) and right (Fig. 1b) and left (Fig. 1c) sides, indicating acute pneumonia. The animal presented severe respiratory failure four hours later and died. Altogether, these results indicate that the cat suffered severe acute respiratory syndrome. Blood samples were collected before death for hematological, serological, and biochemical analyses.

### Hematological, serological, and biochemical analyses

Regarding haematological findings, we found lymphocytopenia (2,2 lymphocytes/mm^3^ in WBC), anemia (RBC = 1, 41 × 10^12^ erytrocytes/L; Haemoglobin = 3,0 g/dL) and severe thrombocytopenia (17 × 10^9^ platelets/L). Regarding serum biochemical markers, increased levels of urea (71.20 mg/dL) and glucose (176 mg/dL) were found. Furthermore, the diagnosis of FIV/FeLV infections performed by serological tests showed a positive result for FeLV, which has been associated with immunosuppression and the hematological disturbances observed.

### Viral RNA detection

Viral RNA was detected in all collected fresh tissues (see Table 1). According to cycle threshold (CT) values, the highest viral load was found in the trachea, followed by the liver, spleen, heart, lungs, kidneys, and intestines. The presence of viral RNA in all these tissue samples indicates a multisystemic viral infection. In addition, the virus was confirmed to be a variant of concern P.1 (Gamma), as shown by genome sequencing. The sequence was deposited at GISAID (EPI_ISL_4565991).

**Table 1:** Presence of SARS-CoV-2 RNA in tissues. The results are expressed as Mean ± SD of CT values for N, E, and RdRP genes

Tissues	CT values of target genes		
	N^a^	E^b^	RdRP^c^
Spleen	23.39 ± 0.16	27.50 ± 0.57	33.72 ± 0.58
Liver	22.46 ± 0.21	22.11 ± 0.36	26.04 ± 0.09
Heart	26.34 ± 0.18	25.43 ± 0.48	28.14 ± 0.26
Lungs	28.16 ± 0.21	28.48 ± 0.41	31.98 ± 0.56
Trachea	16.04 ± 0.1	15.16 ± 0.06	16.98 ± 0.23
Intestines	30.93 ± 0.26	30.87 ± 1,2	36.33 ± 0.39
Kidneys	29.81 ± 0.4	29.27 ± 0.3	33.91 ± 0.93

^a^Nucleoprotein gene

^b^Envelope protein gene

^c^RNA-dependent RNA polymerase (RdRP) gene

### Phylogenetic analysis

**Fig. 2:**
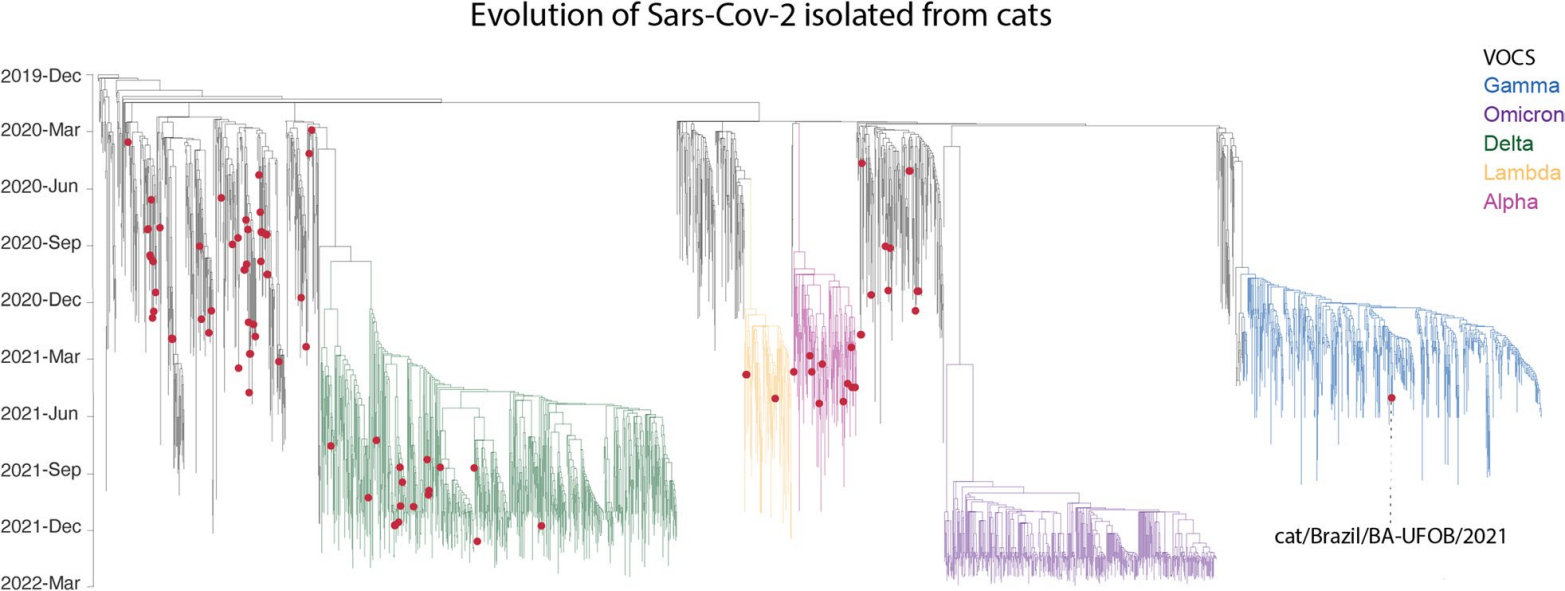
Time tree of the SARS-CoV-2 genomic sequences from the cat-derived sequence and global dataset set of 2,334 high-quality genomes from GISAID was inferred using nextstrain pipeline. The genomes sequences were aligned using MAFFT and a maximum likelihood tree was inferred using IQ-Tree. The ancestral reconstruction and timescale were estimated using augur and treetime. The tree was rooted at Wuhan/WH01/2019 and Wuhan/Hu-1/2019 ancestor.The cat-derived sequences are depicted by dots

As shown in Fig. 2, the newly determined genome clustered within the VOC Gamma. To our knowledge, this is the first description of the Gamma variant lineage sequenced from cats globally. After genome comparison and ancestral mutation reconstruction, no signal of host adaptation was found in EPI_ISL_4565991. All the non-synonymous mutations observed were previously described as the Gamma variant lineage-defining mutations: ORF1a (S1188L, K1795Q), ORF1a (S3675-, G3676-, F3677-), ORF1b (P314L, E1264D), S (L18F, T20N, P26S, D138Y, R190S, K417T, E484K, N501Y, D614G, H655Y, T1027I, V1176F), ORF3a (S253P), ORF8 (E92K), N (P80R, R203K, G204R), ORF9b (Q77E).

### *Post morten* evaluation

**Fig. 3:**
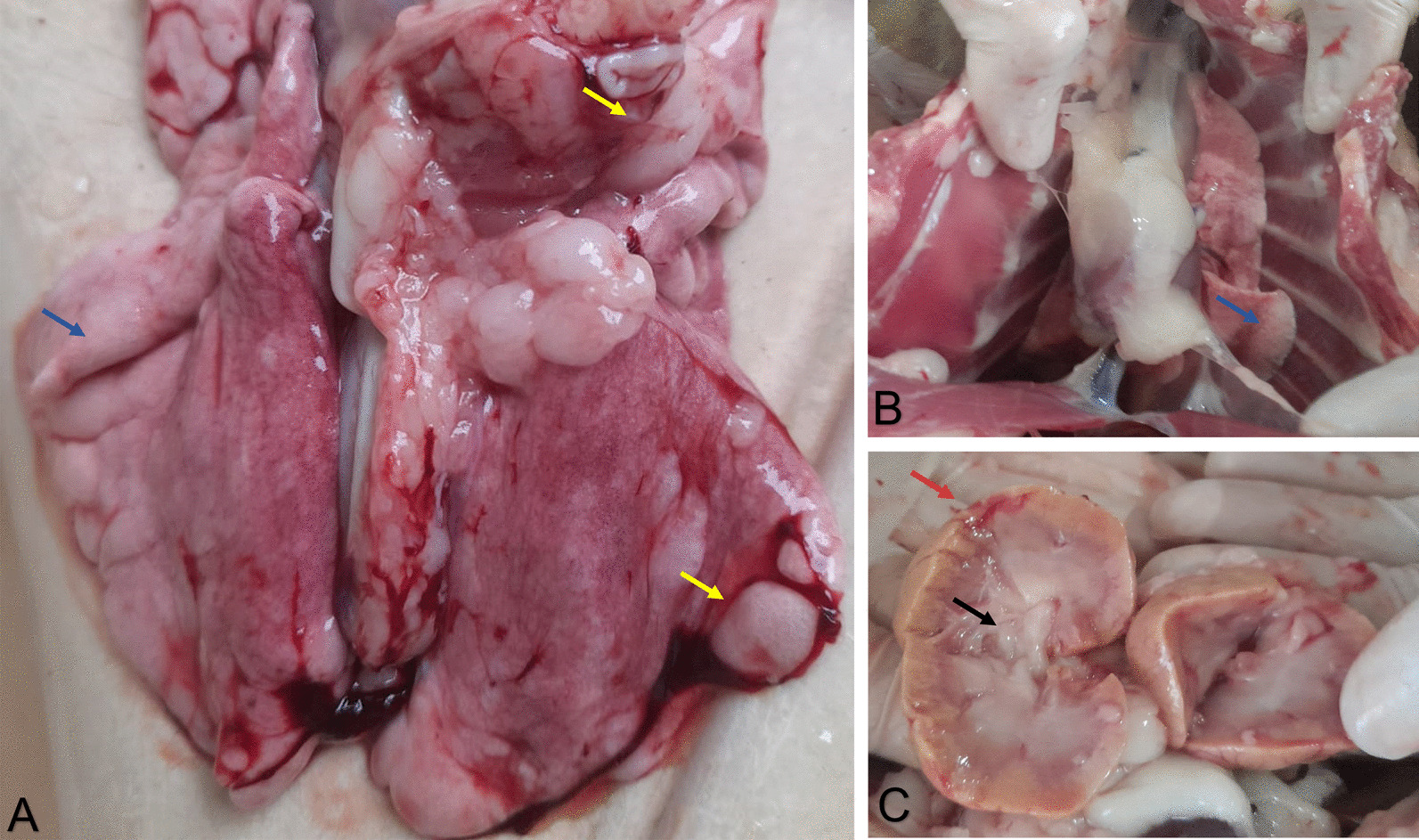
**a, b** Lungs showing atelectasis (blue arrows) and emphysematous areas (yellow arrows). **c** Kidneys showing a cortical decrease (red arrow) and catarrhal-like secretion in the calyx and pelvis (black arrow)

Collapsed and emphysematous areas with air distending the interlobular septa of the lungs were seen in gross pathology (Fig. 3a, b). In addition, the kidneys were shown to be pallid, shorter, irregular, and firm. With a cross-section, the cortical region had a reduced thickness, and there was a secretion with a catarrhal aspect in the calyxes and pelvis (Fig. 3c). These results collectively indicate severe damages in the lungs and kidneys of the feline co-infected by SARS-CoV-2 and FeLV.

### Histopathology

**Fig. 4:**
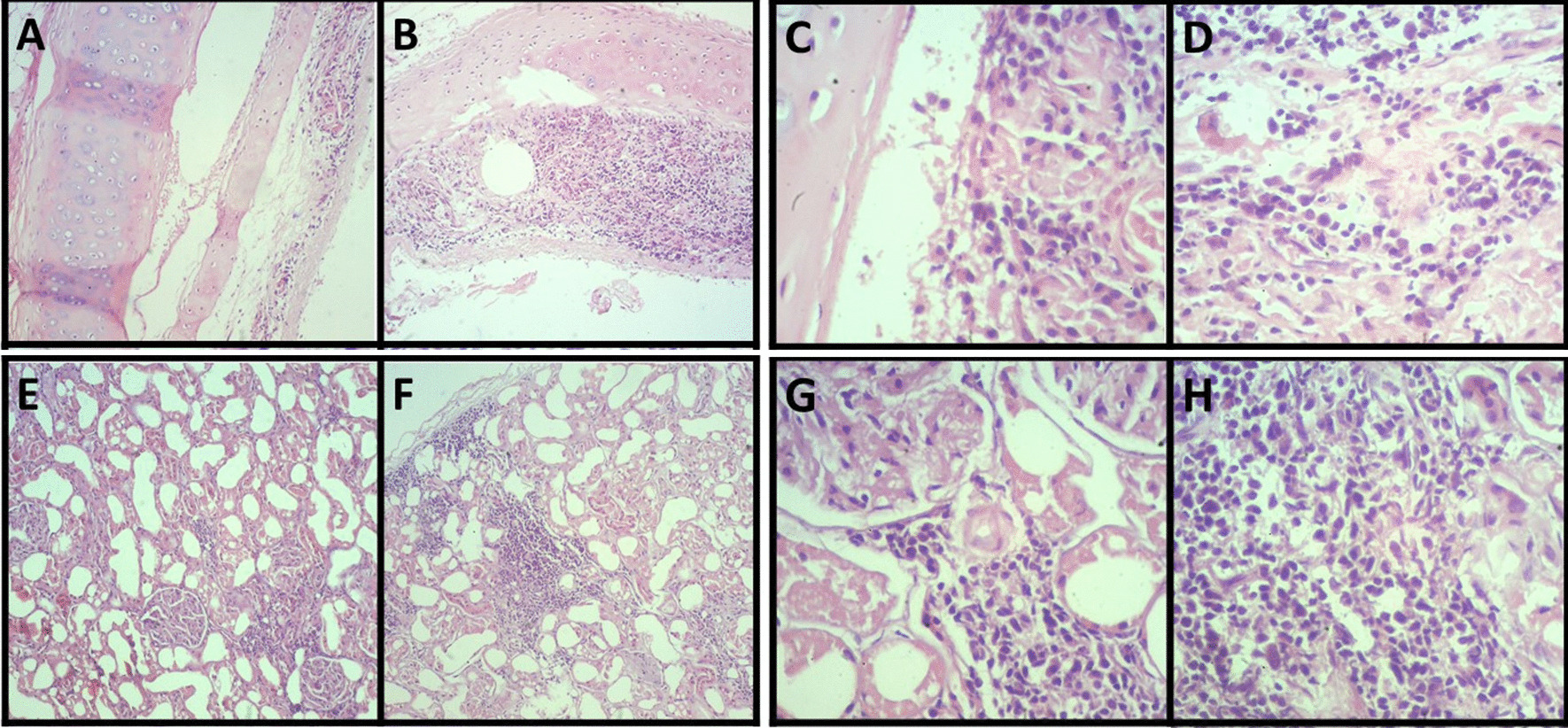
Moderate multifocal lymphoplasmacytic tracheitis. Photomicrography shows a piece of hyaline cartilage without alterations and artifactual distancing from tissue planes and moderate inflammatory infiltrate consisting predominantly of lymphocytes and plasma cells occupying the submucosa of the organ at 40× (**a, b**) and 400× (**c, d**) magnifications. Moderate lymphoplasmacytic interstitial nephritis. Photomicrograph shows the interstitial distribution of inflammation consisting predominantly of lymphocytes and plasma cells at 40× (**e, f**) and 400× (**g, h**) magnifications

**Fig. 5:**
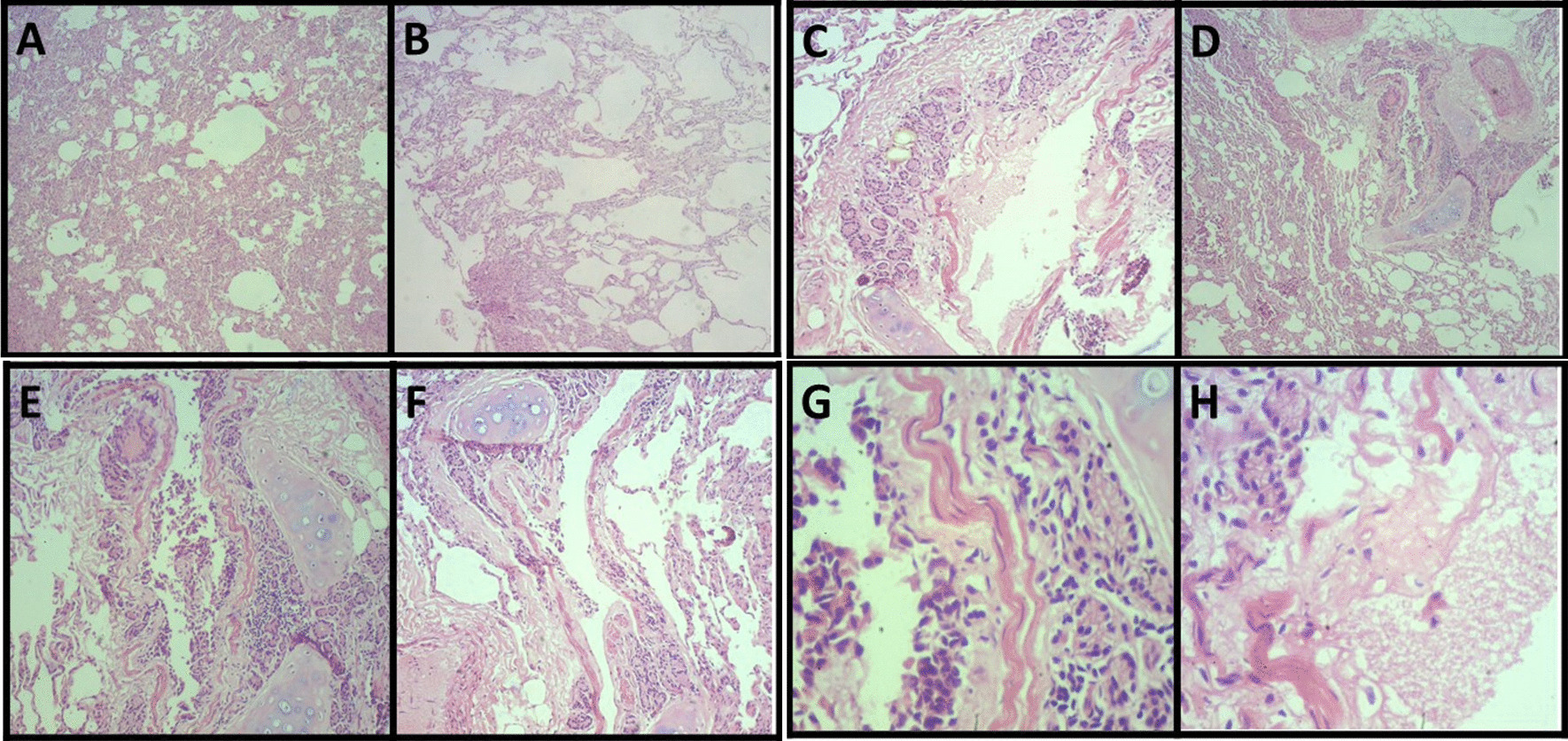
Lung parenchyma exhibits multifocal atelectasis at a magnification of 40× (**a**) and compensatory areas of emphysema at 40× magnification (**b**). Mild lymphoplasmacytic bronchitis. Photomicrography shows a sparse inflammatory infiltrate consisting predominantly of lymphocytes and plasma cells permeating the peribronchial mucous glands at 40× (**a–d**), 100× (**e, f**) and 400× (**g**) magnifications

**Fig. 6:**
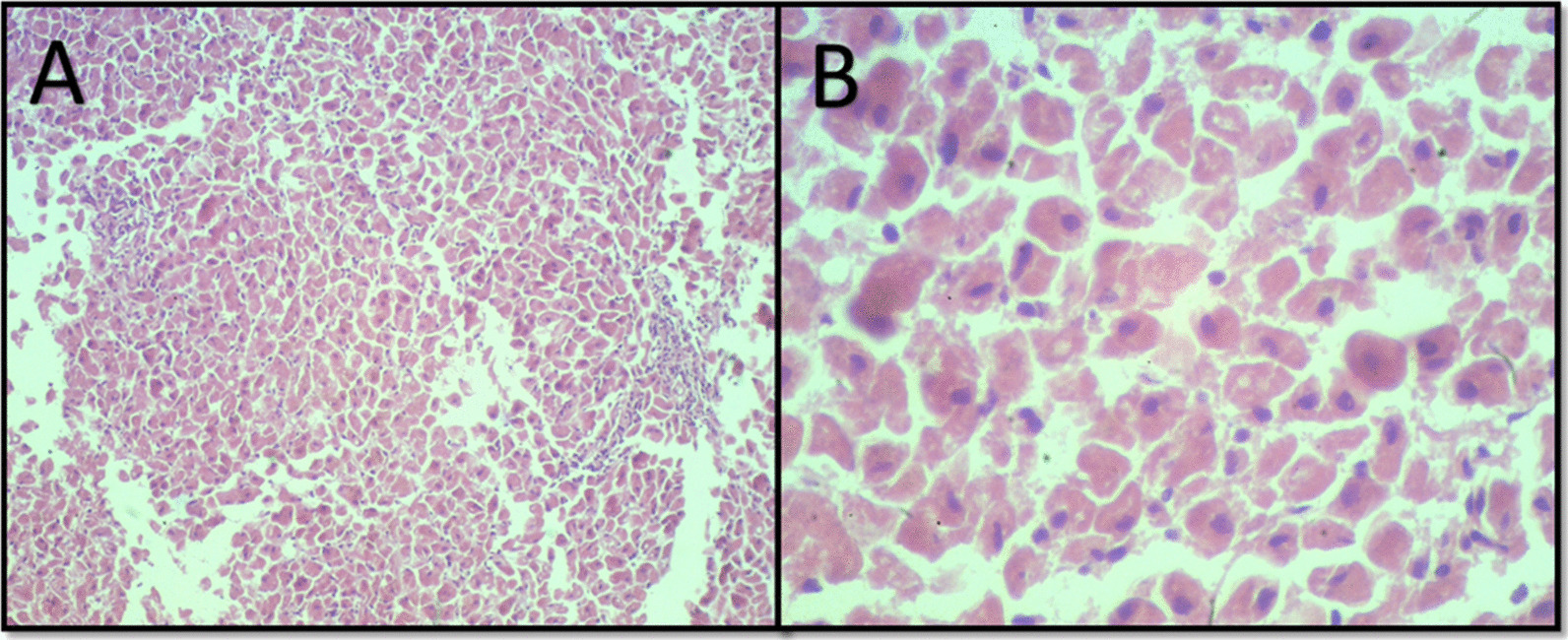
Mild lymphoplasmacytic periportal hepatitis. A sparse inflammatory infiltrate consisting predominantly of lymphocytes and plasma cells is seen at 100× magnification (**a**) and a mild vacuolar degeneration is seen at 400× magnification (**b**)

Histopathological investigation showed specific microscopic changes in the trachea, lungs, liver, and kidneys. Histological sections of the trachea and kidneys revealed multifocal lymphoplasmacytic inflammatory infiltrate, which indicates tracheitis (Fig. 4a–d) and moderate lymphoplasmacytic interstitial nephritis (Fig. 4e–h), respectively (Fig. 4). The lung sections, in turn, revealed multiple areas of atelectasis (Fig. 5a). In addition, compensatory emphysema foci (Fig. 5b) and sparse inflammatory infiltrate associated with the peri bronchial mucous glands were observed (Fig. 5a–g). Furthermore, liver microscopy revealed vacuolar degeneration of hepatocytes, seen in periportal hepatitis. Hepatic lymphoplasmacytic infiltrations were mild (Fig. 6). Collectively, these histopathologic results reinforce that trachea, lungs, liver, and kidneys were severely damaged by SARS-CoV-2 infection.

## Discussion

SARS-CoV-2 probably crossed the species barriers and established humans as new host reservoirs of infection using first an intermediate host [11]. It is possible that animals, including pets, have an unrevealed importance in the spread and even maintenance of COVID-19 worldwide. Events of reverse zoonosis could support such maintenance. The replication of SARS-CoV-2 in animals represents a constant risk of the emergence of new variants with the potential to threaten human health and to challenge the protective efficacy of vaccines currently available [12]. In addition, it is important to highlight that animals are not being vaccinated against COVID-19.

This study presented the first evidence of a cat's multisystemic and lethal SARS-CoV-2 infection. The multisystemic distribution of the virus was confirmed by its detection in several organs, including kidneys and intestines. These findings point to the risk of environmental contamination by urine or feces. In addition, the high viral load seen in the trachea points to the risk of transmission of SARS-CoV-2 to other animals or even humans. Moreover, this is the first report of an animal case infected by the SARS-CoV-2 variant of concern (VOC) P.1 (Gamma) worldwide. Such a VOC caused a dramatic health crisis in Brazil and other countries of South America [13–15] due to its high infectivity and immune escape capacity [16]. We highlight that many infections caused by VOC P.1 were registered in Bahia at the time of the cat infection, which moved the state health department to suspend non-essential trips [17].

It is also important to highlight that the cat was FeLV-positive. Such a co-infection may have increased the severity of the COVID-19 seen in that animal, which involved an evident severe acute respiratory syndrome. The FeLV [11] is a retrovirus that infects cats. Contaminated saliva or nasal secretions transmit the virus, which is capable of compromising the animal`s immune system [12]. In this study, we observed hematological disturbances related to FeLV infection. In general, such pathological signs are accompanied by immunosuppression [13].

We hypothesize that beyond the FeLV/SARS-CoV-2 co-infection led to a lethal severe acute respiratory syndrome, it also enhanced the viral load in organs that can contribute to environmental contamination and direct transmission of the coronavirus. In addition, such a co-infection may represent a threat for FeLV-positive cats and should be carefully investigated in cases of clinical suspects. In this context, detailed pathologic data presented here should be used to recognize and manage cats that are more susceptible to SARS-CoV-2 infection.

## Conclusion

Our study indicates that replication of SARS-CoV-2 in high loads occurs in FeLV-positive cats. This represents a constant risk of the emergence of new variants with the potential to threaten human health and challenge vaccines' protective efficacy. Therefore, pets should be surveilled regarding SARS-CoV-2 infection. In addition, the need for a strong and engaged One Health approach in all areas of the world is essential to tackle SARS-CoV-2 re-emergence.

## Supplementary Information

**Additional file 1**.** Supplementary Table 1**. GISAID acknowledgment table including 2,334 genome sequences used in our study.

## Data Availability

Genomic data was deposited to GISAID. Other data will be provided under request.
